# Effects of *Ureaplasma urealyticum* infection on semen quality and sperm morphology

**DOI:** 10.3389/fendo.2023.1113130

**Published:** 2023-03-06

**Authors:** Fu Xianchun, Fang Jun, Dai Zhijun, Hong Mingyun

**Affiliations:** Centre for Reproductive Medicine, Affiliated Maternity and Child Health Hospital of Anhui Medical University, Anhui Province Maternity and Child Health Hospital, Hefei, Anhui, China

**Keywords:** Ureaplasma urealyticum, semen quality, sperm morphology, male genitourinary infection, infertile men

## Abstract

**Introduction:**

*Ureaplasma urealyticum* (*U. urealyticum*) infection is primarily associated with damage to male fertility through its effects on male sperm parameters. However, its effects on sperm semiological variables remain unclear. Therefore, this study aimed to investigate whether *U. urealyticum* infection was associated with semen quality and sperm morphology.

**Methods:**

From 2019 to 2021, this cross-sectional study analyzed infective pathogens and semen variables in 1064 males (22–30 years old) recruited from our reproductive center and the general public. Routine semen parameters and normal sperm morphology rate were analyzed using methods outlined by the World Health Organization. The associations between semen quality, sperm morphology, and *U. urealyticum* infection were studied using general linear models.

**Results:**

The participants were categorized into three groups: (i) *U. urealyticum* infection (n=328), (ii) non-*U. urealyticum* infection (including males with urogenital tract infection symptoms but no *U. urealyticum* detected in their semen samples, n=377), and (iii) normal volunteers (males without symptoms of urogenital tract infection and no pathogens detected in semen samples, n=359). *U. urealyticum* in semen samples was observed to be associated with lower sperm concentrations (p<0.001) and a lower ratio of anterograde motile spermatozoa (p<0.001). Semen cultures positive for *U. urealyticum* were associated with lower normal sperm morphology (p<0.001) compared to semen cultures negative for *U. urealyticum*.

**Conclusion:**

This study shows the importance of proper investigations for *U. urealyticum* during routine clinical examinations and diagnoses of males with infertility.

## 1 Introduction

Recently, increasing attention has been paid to male reproductive health. Many studies have analyzed factors influencing sperm quality, such as obesity, lifestyle, working conditions, diet culture, congenital and heredity factors, and urogenital infections (1). *Ureaplasma urealyticum* (*U. urealyticum*) is considered a common pathogen of urogenital system-related infections that affect sperm and semen quality. Inflammatory reactions and factors caused by *U. urealyticum* infection likely affect the surrounding environment and normal sperm function. Moreover, *U. urealyticum* infection is considered a common infection that contributes to changes in the internal structure of sperm cells. Studies have reported that current clinical *mycoplasma* infections in the urogenital system of infertile males are mainly caused by *U. urealyticum* (2). The association between *U. urealyticum* infection and male infertility has been extensively studied; however, controversies persist regarding whether *U. urealyticum* infection could lead to male infertility. Similarly, the association between *U. urealyticum* infection in the reproductive tract and semen parameters is controversial (3).

Males of childbearing age are likely to suffer from *U. urealyticum* infection due to their active sexual life. Usually, urogenital symptoms of *U. urealyticum* infection are similar to those of bacterial infections in the urinary tract. In addition to being related to a decline in some sperm parameters (4), *U. urealyticum* is often considered the main cause of male sperm cell distortion (5). It can integrate with the head of sperm cells and cause reduced sperm motility and individual gene mutation by working as a strong distortion agent (6). Another form of sperm distortion caused by *U. urealyticum* infection is sperm cell deformity (7). This deformity likely develops distortions such as a decrease in the sperm’s overall antioxidant capacity and flaws emerging in DNA repair (8). Therefore, a positive correlation between *U. urealyticum* infection and semen parameters suggests that *U. urealyticum* infection is a possible index of male infertility (9).

*U. urealyticum* is the main causative organism of male genital tract infections. However, other pathogens such as bacteria, *Chlamydia trachomatis* (CT), *Neisseria gonorrhea* (NG), molds, and *trichinosis* might also cause genital tract infections (10) at relatively low incidence rates. Hence, a previous study fully summarized the effect of *U. urealyticum* infection on semen by collecting and analyzing *U. urealyticum* and semen parameters in male patients with infertility (11) compared with data from normal patients.

Previous studies have reported that *U. urealyticum* infection is associated with normal sperm morphology rate (12), while others have not observed any relationship between the two (11). In addition, the outcome seems discordant with the genre of *U. urealyticum* infection for which such an association was discovered (13). Moreover, evidence shows that *U. urealyticum* infection likely results in DNA chain fractures of human semen cells. However, no association was detected between *U. urealyticum* infection and the sperm fragmentation index (DFI) evaluated by sperm chromatin structure assay (SCSA) (13).

Therefore, due to the controversies in current research, we aimed to study the incidence and impact of male genital *U. urealyticum* infection in homogeneous males with infertility.

## 2 Materials and methods

### 2.1 Study design and participants

Data for this study were collected from a randomized controlled trial that investigated the association of *U. urealyticum*, compared with semen parameters, as a pathogen in urogenital tract infection.

This study adopted the definition of male infertility by the World Health Organization (WHO, 5th edition), which is the inability of a male to fertilize his partner after >12 months of cohabitation with normal sexual intercourse and no contraceptive measures (10). Based on this definition, this study included 705 males aged 20–30 years with infertility who visited the reproductive center of Anhui Province Maternal and Child Health Hospital (Hefei, China) from January 2019 to May 2021. The study also included 359 young males aged 22–30 years from the general public who were permanent residents in Hefei, China. Males from the general public were recruited by (i) routine physical examinations before pregnancy (n=213) and (ii) publicity on campus (n=146). All 1064 recruited males signed the informed consent form, completed questionnaires about whether they had urogenital infection symptoms, collected and sent semen samples the same morning, and were paid 500 RMB as a reward for participation. The recruited males (i) had been residents in Hefei City for >5 years and were of Han ethnicity; males conforming to these criteria were asked to participate in the program. Data collection and investigation on *U. urealyticum* infection and male fertility were approved by the ethics Review Committee of Anhui Province Maternal and Child Health Hospital. Furthermore, this study was conducted in accordance with the Declaration of Helsinki.

Data collected from the 1064 males were as follows (1): duration of the abstinence period before semen sample delivery (2), semen routine parameters and normal sperm morphology rate, and (3) pathogens in the semen. Based on the results of laboratory cultures, the participants were divided into three groups: (i) *U. urealyticum* infection, including males with *U. urealyticum* detected in their semen samples, n=328), (ii) non-*U. urealyticum* infection, including males with urogenital tract infection symptoms but no *U. urealyticum* detected in their semen samples, n=377), and (iii) normal volunteers, including males with no urogenital tract infection symptoms and no pathogens detected in their semen samples, n=359).

### 2.2 Sample collection

Samples of human semen were collected by masturbation during an abstinence period, which was standardized to be 3–5 days in line with the WHO 2010 guidelines (14). The exact duration of the abstinence period was recorded. All males were instructed about semen and genital specimen collection, according to the national standard of reproductive laboratories, before masturbating to minimize microbial contamination during semen extraction (15). Each semen sample was poured into a sterile glass cup and liquefied in specific incubators at 37°C. When the semen sample was entirely liquefied, a volume ≥2.0 ml was required for this study. Each semen sample was used for three purposes: (i) routine semen analysis, (ii) analyzing the ratio of normal sperm morphology, and (iii) for pathogen testing.

### 2.3 Routine semen analysis

Routine semen analysis was performed according to the WHO Laboratory Manual for the Examination and Processing of Human Semen (5th edition) (16). Semen volume, sperm concentration, and percentage of forward movement (PR%) were analyzed using an automatic semen quality computer-aided analysis system (Spanish SCA SCOPE Company). The image acquisition module and frame image were implemented using the following parameters: most sperm concentrations were collected at 20 Hz, while higher concentrations were collected at 7 Hz; collection intermission, 3 ms; maximum velocity of sperm motility, 200 um/s; sperm motility index, and straight-line velocity. The grayscale threshold values were formulated to determine the spermatozoa and eliminate non-sperm components. Then, sperm parameters were analyzed and compared per the thresholds formulated for the semen assay.

Semen volume (mL), viscosity, liquefaction degree, and appearance were measured manually according to standard operating procedure (SOP) specifications, and the pH was tested using a litmus test paper. Next, 10 µL of each semen sample was drawn using a sampling gun on a sperm counting plate (Israel Self Medical Instruments Company). The sperm concentration (×10^6/^ml) was obtained using an automatic semen quality computer-aided analysis system (Beijing Zhongke Hengye Co., Ltd.). Other data collected were the rates of forward sperm (%), non-forward sperm (%), inactive sperm (%), survival rate (%), average path velocity (um/s), linear motility (%), wobble (%), linear (%), and straightness (%), as well as curve velocity (um/s), the amplitude of lateral head display (um), beat cross frequency (Hz), mean angular displacement, and straight-line velocity (um/s).

Sperm smear samples were stained using the new Pasteur staining method. The percentages of normal sperm, head defects, middle and main segment defects, excessive residual cytoplasm, and round cells were observed under an oil microscope. The operation was strictly performed following the SOP.

#### 2.3.1 Ratio of sperm normal morphology assessment

Semen smears were dyed using a Diff-Quick reagent (MICROPTIC S.L. Co., Barcelona, Spain) to analyze the ratio of normal sperm morphology (14). Briefly, 10 µL of semen sample was diffused onto a glass slide and then air-dried at an indoor temperature for more than 10 min. The smeared glass slide was stained and analyzed using a high-power microscope (BX53M; Olympus, Qingdao, China) magnified 1000 times. In line with the 2010 WHO guidelines, a spermatozoon with a malformed head, middle, and tail parts was recorded as teratozoospermia. The sperm deformity index (SDI) was calculated as the ratio of deformed sperm cells to the total number of sperm cells (17). For each sperm sample, an outnumber of 200 spermatozoa (or the entire spermatozoa if the glass slide held <200 sperm cells) was calculated using a double-blind approach. Additionally, the ratio of spermatozoa with deformities or normal morphology was calculated.

### 2.4 Detection of pathogenic microorganisms

All semen samples from the study population were evaluated for *U. urealyticum*. *U. urealyticum* was detected using polymerase chain reaction (PCR) with specific kits (Daan Gene Co. Ltd., Guangzhou, China). A standard curve was formulated for each test based on the kit contents. Concentrations of *U. urealyticum* DNA ≥500 copies/ml were considered positive (18). *U. urealyticum* was analyzed under high-quality control in the professional reproductive laboratory of Anhui Province Maternal and Child Health Hospital, approved by the China Food and Drug Administration (19).

### 2.5 Statistical analysis

Patients’ age, semen volume, pH value, liquefaction time, sperm concentration, forward moving sperm, non-forward moving sperm, viability, average path velocity, curve velocity, the amplitude of lateral head display, beat cross frequency, mean angular displacement, straight-line velocity, wobble, linear, straightness, as well as the rates of normal morphology, head deformity, and middle and main segment deformity, and round cell number, were measured and distributed normally, expressed as mean ± standard deviation (x ± s). One-way ANOVA was used to compare the differences between groups. Viscosity was a classified variable, and non-parametric tests were used for statistical analyses. *Post-hoc* comparisons were analyzed using Tukey’s test, where the result manifested a significant main effect, and the percent relative effects and 95% confidence intervals (CIs) were calculated. Statistically significant differences were considered at p-values <0.05, and SPSS 20.0 was used for all statistical analyses.

## 3 Results

### 3.1 Grouping of the study population

**Table 1** shows normal standards for male semen parameters, such as semen volume, sperm density, percentage of sperm forward movement, the normal sperm morphology rate, pH, and sperm activation and survival rates. According to the results of microbial culture tests, **Table 2** shows that the semen samples of 328 (30.83%) males had microbial cultures positive for *U. urealyticum*. Approximately 377 (35.43%) males were considered to have symptoms of urogenital infections based on their responses to the questionnaires; however, *U. urealyticum* was not detected in their semen samples. In contrast, 359 (33.74%) males were observed to have no urogenital infection symptoms, and *U. urealyticum* was not detected in their semen samples.

**Table 1:** Normal standards on male semen quality parameters.

Parameters	Lower limit of the reference value
Semen volume (ml)	1.5 (1.4–1.7)
Sperm density (× 10^6/^ml)	15 (12–16)
Sperm activation rate (%)	40% (38–42%)
Percentage of sperm forward movement (%)	32% (31–34%)
Sperm survival rate (%)	58% (55–63%)
Normal rate of sperm morphology (%)	4% (3–4%)
pH	7.2

**Table 2:** Male genital tract pathogen infection.

variables	number (n)	rate (%)
*U. urealyticum* infection group	328	30.83
Non-*U. urealyticum* infection group	377	35.43
Normal volunteers group	359	33.74

### 3.2 *U urealyticum* infection and semen routing parameters

Semen pH and liquefaction time were significantly higher in the *U. urealyticum* infection group than in the non-*U. urealyticum* infection and normal volunteers groups (7.28 ± 0.02 vs. 7.15 ± 0.03 vs. 7.22 ± 0.03; p=0.003 and 31.87 ± 1.41 vs. 30.72 ± 1.32 vs. 30.81 ± 1.40; p<0.001, respectively) (**Table 3**). In contrast, the sperm density and ratio of anterograde motile spermatozoa decreased significantly (67.52 ± 33.15 vs. 84.22 ± 41.85 vs. 73.88 ± 25.09; p<0.001 and 36.48 ± 12.70 vs. 38.97 ± 6.82 vs. 43.31 ± 8.78; p<0.001, respectively) (**Table 3**).

**Table 3:** Correlation between male genital tract pathogen detection and routine semen quality indicators (mean ± standard deviation).

Variables	*U. urealyticum* infection	non-*U. urealyticum* infection	normal volunteers	F-value	p-value
Age (y)	34.94 ± 7.48	33.87 ± 6.41	33.19 ± 6.36	1.06	0.377
Volume (ml)	2.90 ± 1.17	2.89 ± 0.65	2.35 ± 0.58	1.59	0.175
pH	7.28 ± 0.02	7.15 ± 0.03	7.22 ± 0.03	4.01	0.003
Liquefaction time (min)	31.87 ± 1.41	30.72 ± 1.32	30.81 ± 1.40	7.47	<0.001
Density (× 10^6/^ml)	67.52 ± 33.15	84.22 ± 41.85	73.88 ± 25.09	23.45	<0.001
Forward direction (%)	36.48 ± 12.70	38.97 ± 6.82	43.31 ± 8.78	36.70	<0.001
Non-forward (%)	49.96 ± 9.24	31.38 ± 6.95	39.88 ± 3.69	37.60	<0.001
Inactive (%)	49.96 ± 15.15	31.38 ± 6.39	39.88 ± 11.50	162.69	<0.001
Activity rate (%)	59.19 ± 13.38	68.79 ± 6.69	65.17 ± 10.01	88.77	<0.001
VAP (um/s)	11.14 ± 4.03	12.02 ± 2.85	22.21 ± 25.14	181.80	<0.001
VCL (um/s)	12.67 ± 4.05	14.22 ± 2.63	16.41 ± 4.26	18.95	<0.001
ALH (um/s)	0.66 ± 0.18	0.75 ± 0.06	0.74 ± 0.15	1.24	0.291
BCF (Hz)	12.79 ± 1.02	13.87 ± 1.41	12.87 ± 1.41	12.03	<0.001
MAD (um/s)	99.63 ± 10.61	96.12 ± 7.36	94.22 ± 4.23	200.41	<0.001
VSL (um/s)	7.79 ± 2.79	8.63 ± 2.51	10.57 ± 2.94	22.98	<0.001
sperm motility (%)	40.40 ± 13.07	42.98 ± 5.74	47.21 ± 9.12	39.23	<0.001
WOB (%)	68.45 ± 12.46	71.61 ± 7.55	76.69 ± 9.25	105.19	<0.001
LIN(%)	44.61 ± 9.14	45.68 ± 6.27	51.14 ± 7.03	63.46	<0.001
STR(%)	60.51 ± 6.33	60.93 ± 6.41	63.87 ± 4.13	42.52	<0.001
Viscosity				0.737	0.565

ALH, amplitude of lateral head display; BCF, beat cross frequency; LIN, linear; MAD, mean angular displacement; STR, straightness; VAP, average path velocity; VCL, curve velocity; VSL, straight line velociyt; WOB, wobble.

p<0.05 was considered statistically significant.

Viscosity is a classified variable, and a non-parametric test was used for statistical analysis.

### 3.3 *U urealyticum* infection and sperm morphology

*U. urealyticum* infection was significantly associated with lower ratios of sperm with normal morphology (7.39 ± 3.23 vs. 10.11 ± 3.61 vs. 7.98 ± 4.51; p<0.001), compared with semen males without *U. urealyticum* infection (**Table 4**).

**Table 4:** Correlation between male genital tract pathogen detection and sperm morphology (mean ± standard deviation).

Variables	*U. urealyticum* infection	non-*U. urealyticum* infection	Normal volunteers	F-value	p-value
Normal form(%)	7.39 ± 3.23	10.11 ± 3.61	7.98 ± 4.51	18.87	<0.001
Head defect(%)	89.43 ± 3.64	84.59 ± 4.97	89.65 ± 5.43	116.22	<0.001
Middle segment defect(%)	21.14 ± 6.83	23.78 ± 4.76	19.93 ± 3.30	20.46	<0.001
Main section defect(%)	16.41 ± 5.30	16.63 ± 3.70	14.65 ± 2.53	3.21	0.011
Excess cytoplasm(%)	1.38 ± 0.95	1.57 ± 0.73	1.07 ± 0.73	0.67	0.562
Round cell(%)	2.46 ± 1.73	3.57 ± 1.73	2.57 ± 0.73	9.03	<0.001

p<0.05 was considered statistically significant.

## 4 Discussion

Due to the implementation of the two-child policy amendment of China’s Population and Family planning Law, the number of males who want to reproduce offspring has increased significantly (20). In addition, *U. urealyticum* infection of the genital tract has been confirmed as an important risk factor for male infertility (21). Hence, it is important to determine whether treating male *U. urealyticum* infections is necessary to improve male reproductive function. Several studies have shown that *U. urealyticum* infection in the male genital tract negatively affects semen quality (22). This study showed that *U. urealyticum* infection in the male genital tract significantly affected the pH value, liquefaction time, concentration, and motility of forward-moving sperm in semen samples. These results are consistent with those of most previous studies.

Data collected in this study showed that *U. urealyticum* infection is common in young males (30.83%). This result was higher than that in most studies (23), which could probably be ascribed to either this study’s detection method or regional differences.

After *U. urealyticum* infection in the male genital tract, the number, concentration, activity, and survival rate of sperm cells were significantly reduced. Forward sperm and survival rates are the most important parameters for semen quality and directly affect the sperm-egg combination (24), and *U. urealyticum* infection impacts sperm concentration and forward movement (25). Infection of the reproductive tract leads to male spermatozoa acclimatization and interferes with sperm maturation in convoluted tubules. Furthermore, mixed infections had a more significant effect on routine semen parameters than other pathogens. The number of non-forward motile spermatozoa increases significantly after infection with mixed pathogens (26). The effect of *U. urealyticum* infection on sperm cells may be due to its ability to adhere to sperm cells and integrate to affect cellular interactions directly. Sperm vitality, motility, morphology, cellular integrity, and molecular structure also deteriorate due to the development of protective immunity against genital infection by the host (population sensitivity to microbial agents).

Compared with semen samples without pathogens, *U. urealyticum* infection in semen samples significantly increases the rate of sperm malformation (27). When the ratio of normal sperm cells is <4%, it affects the sperm-egg combination and reduces the probability of pregnancy. *U. urealyticum* infection affects sperm morphology by integrating with sperm cells. It assaults sperm cells in the middle of the head and the area behind the chromosome. The sperm membrane at the adsorption site is damaged or even severely destroyed, making the sperm from streamlined to “bloated,” with abnormal morphology. The tail becomes angled and curled, and some sperm are agglutinated. After *U. urealyticum* infection, the rate of sperm malformation increases, and abnormal morphology, such as cusps, big heads, double heads, and tailless, increases (28). It can also stimulate the body to produce various inflammatory mediators that damage sperm morphology (29).

Compared with the uninfected group, males with *U. urealyticum* infection had higher middle segment deformity, round cells, and excessive cytoplasm, which could reduce the ratio of normal sperm and might cause dyspepsia and male infertility (30). Co-incubation of *U. urealyticum* and sperm led to a significant decrease in the number of motile sperm cells and premature sperm death. *U. urealyticum* lipopolysaccharide is the main cause of sperm apoptosis, and lipopolysaccharide levels in semen affect sperm quality and function (31). In this study, the normal morphology rate of males infected with *U. urealyticum* decreased more significantly than that of those infected with other pathogens and the normal volunteers. Therefore, male genital tract infection was closely related to male infertility (32). However, the number of other pathogenic specimens identified was small and should be expanded for further studies.

This study observed that male genital tract infection with *U. urealyticum* is significantly associated with conventional semen parameters (including morphology). The detection and treatment of *U. urealyticum* are recommended for males with infertility to improve their semen quality.

## Data availability statement

The original contributions presented in the study are included in the article/supplementary material. Further inquiries can be directed to the corresponding author.

## Ethics statement

Written informed consent was obtained from the individual(s) for the publication of any potentially identifiable images or data included in this article.

## Author contributions

Each author contributed to the search process. The experiments were conceived and designed by FX, FJ, DZ. Carry out experiments: FX, FJ, DZ. HM. Analysis data: FX. Compilation manual: FJ. The manual has been strictly revised, and the final version has been completed/corrected: FX, FJ, HM. All authors contributed to the article and approved the submitted version.

## References

[1] Al-Sweih NA Al-Fadli AH Omu AE Rotimi VO. Prevalence of chlamydia trachomatis, mycoplasma hominis, mycoplasma genitalium, and ureaplasma urealyticum infections and seminal quality in infertile and fertile men in Kuwait. J Androl (2011) 33(6):1323–9. doi: 10.2164/jandrol.111.013821 22052774

[2] Beeton ML Payne MS Jones L. The role of ureaplasma spp. in the development of nongonococcal urethritis and infertility among men. Clin Microbiol Rev (2019) 32(4):e00137-18. doi: 10.1128/CMR.00137-18 31270127 PMC6750135

[3] Gao E-S Wu J-Q Liang C-L Chen X-K. 501: The relationship between ureaplasma urealyticum infection in the genital tract and semen quality. Am J Epidemiol (2005) 161(Supplement_1):S126–6. doi: 10.1093/aje/161.supplement_1.s126

[4] Gdoura R Kchaou W Chaari C Znazen A Keskes L Rebai T. Ureaplasma urealyticum, ureaplasma parvum, mycoplasma hominis and mycoplasma genitalium infections and semen quality of infertile men. BMC Infect Dis (2007) 7(1):129. doi: 10.1186/1471-2334-7-129 17988404 PMC2194714

[5] Huang C Zhu HL Xu KR Wang SY Fan LQ Zhu WB. Mycoplasma and ureaplasma infection and male infertility: a systematic review and meta-analysis. Andrology (2015) 3(5):809–16. doi: 10.1111/andr.12078 26311339

[6] Lafuente R García-Blàquez N Jacquemin B Checa MA. Outdoor air pollution and sperm quality. Fertil Steril (2016) 106(4):880–96. doi: 10.1016/j.fertnstert.2016.08.022 27565259

[7] Le MT Nguyen TLN Le DD Ngo TVQ Nguyen ATC Nguyen BH. Is genital tract infection related to tubal diseases in infertile Vietnamese women? J Infect Develop Countries (2019) 13(10):906–13. doi: 10.3855/jidc.11632 32084021

[8] Lee JS Kim KT Lee HS Yang KM Seo JT Choe JH. Concordance of ureaplasma urealyticum and mycoplasma hominis in infertile couples: Impact on semen parameters. Urology (2013) 81(6):1219–24. doi: 10.1016/j.urology.2013.02.044 23602797

[9] McGill JJ Baker K Starks C Barazani Y Sabanegh E. The effects of pyospermia and ureaplasma urealyticum culture positivity on semen quality in subfertile men. Fertil Steril (2013) 100(3):S216. doi: 10.1016/j.fertnstert.2013.07.1302

[10] Moridi K Hemmaty M Azimian A Fallah MH Khaneghahi Abyaneh H Ghazvini K. Epidemiology of genital infections caused by mycoplasma hominis, m. genitalium and ureaplasma urealyticum in iran; a systematic review and meta-analysis study (2000–2019). BMC Public Health (2020) 20(1):1020. doi: 10.1186/s12889-020-08962-5 32600306 PMC7322857

[11] Zinzendorf NY Kouassi-Agbessi BT Lathro JS Don C Kouadio L Louko YG. Ureaplasma urealyticum or mycoplasma hominis infections and semen quality of infertile men in Abidjan. J Reprod Contraception (2008) 19(2):65–72. doi: 10.1016/s1001-7844(08)60008-5

[12] Ozarda Y Ichihara K Barth JH Klee G. Protocol and standard operating procedures for common use in a worldwide multicenter study on reference values. Clin Chem Lab Med (2013) 51(5):1027. doi: 10.1515/cclm-2013-0249 23633469

[13] Peerayeh SN Yazdi RS Zeighami H Razeghi M. Ureaplasma urealyticum in semen of men with varicocele. Lab Med (2008) 39(4):215–7. doi: 10.1309/k4nvb47rgfexa9bm

[14] Riley DE Samadpour M Krieger JN. Detection of variable DNA repeats in diverse eukaryotic microorganisms by a single set of polymerase chain reaction primers. J Clin Microbiol (1991) 29(12):2746–51. doi: 10.1128/jcm.29.12.2746-2751.1991 PMC270426 1757544

[15] Wang Y Han XD Hou YY Chen JX. Ureaplasma urealyticuminfection related to seminal plasma immunosuppressive factors, semen ph and liquefaction duration. Arch Androl (2005) 51(4):267–70. doi: 10.1080/014850190923413 16036633

[16] Wang Y Liang C-L Wu J-Q Xu C Qin S-X Gao E-S. Do ureaplasma urealyticum infections in the genital tract affect semen quality? Asian J Androl (2006) 8(5):562–8. doi: 10.1111/j.1745-7262.2006.00190.x 16752003

[17] Wang Y Wu Z-W Zhang L-F Wu X-K Yi L Han X-D. Effects of ureaplasma urealyticum infection on the male reproductive system in experimental rats. Andrologia (2010) 42(5):297–301. doi: 10.1111/j.1439-0272.2009.00993.x 20860627

[18] Yoshida T Maeda S-I Deguchi T Ishiko H. Phylogeny-based rapid identification of mycoplasmas and ureaplasmas from urethritis patients. J Clin Microbiol (2002) 40(1):105–10. doi: 10.1128/jcm.40.1.105-110.2002 PMC120092 11773101

[19] Zhang Q-F Zhang Y-J Wang S Wei Y Li F Feng K-J. The effect of screening and treatment of ureaplasma urealyticum infection on semen parameters in asymptomatic leukocytospermia: a case–control study. BMC Urol (2020) 20(1):165. doi: 10.1186/s12894-020-00742-y 33092572 PMC7579809

[20] Zhou YH Ma HX Shi XX Liu Y. Ureaplasma spp. in male infertility and its relationship with semen quality and seminal plasma components. J Microbiol Immunol Infect (2018) 51(6):778–83. doi: 10.1016/j.jmii.2016.09.004 28739435

[21] Zhu X Li M Cao H Yang X Zhang C. Epidemiology of ureaplasma urealyticum and mycoplasma hominis in the semen of male outpatients with reproductive disorders. Exp Ther Med (2016) 12(2):1165–70. doi: 10.3892/etm.2016.3409 PMC4950889 27443698

[22] EzzEl-Din AM Gaber HD Kamal DT. Chlamydia trachomatis infection: its relation to semen parameters and sperm DNA integrity. Egypt J Immunol (2021) 28:290–8. doi: 10.55133/eji.280430 34882378

[23] Jaworek H Koudelakova V Oborna I Zborilova B Brezinova J Ruzickova D. Impact of human papillomavirus infection on semen parameters and reproductive outcomes. Reprod Biol Endocrinol (2021) 19:156. doi: 10.1186/s12958-021-00840-y 34627284 PMC8501609

[24] Liu KS Mao XD Pan F An RF. Effect and mechanisms of reproductive tract infection on oxidative stress parameters, sperm DNA fragmentation, and semen quality in infertile males. Reprod Biol Endocrinol (2021) 19:97. doi: 10.1186/s12958-021-00781-6 34183027 PMC8237428

[25] Perez-Soto E Fernandez-Martinez E Oros-Pantoja R Medel-Flores O Miranda-Covarrubias JC Sanchez-Monroy V. Proinflammatory and oxidative stress states induced by human papillomavirus and chlamydia trachomatis coinfection affect sperm quality in asymptomatic infertile men. Med (Kaunas) (2021) 57(9):862. doi: 10.3390/medicina57090862 PMC8466842 34577786

[26] Tam Le M Nguyen Nguyen D Bach Nguyen H Quynh Tram Ngo V Quoc Huy Nguyen V. Ureaplasma urealyticum and mycoplasma genitalium detection and sperm quality: A cross-sectional study in Vietnam. Int J Reprod BioMed (2021) 20:185–94. doi: 10.18502/ijrm.v20i3.10710 PMC9099368 35571499

[27] Wang Z Liu W Zhang M Wang M Wu H Lu M. Effect of hepatitis b virus infection on sperm quality and outcomes of assisted reproductive techniques in infertile males. Front Med (Lausanne) (2021) 8:744350. doi: 10.3389/fmed.2021.744350 34796185 PMC8592897

[28] Bai S Li Y Hu MH Wu L Shui LJ Wang XH. Association of sexually transmitted infection with semen quality in men from couples with primary and secondary infertility. Asian J Androl (2022) 24:317–22. doi: 10.4103/aja202164 PMC9226702 34782548

[29] Hulse L Palmieri C Beagley KW Larkin R Keeley T Gosalvez J. Investigation of pathology associated with chlamydia pecorum infection in the male reproductive tract, and the effect on spermatogenesis and semen quality in the koala (Phascolarctos cinereus). Theriogenology (2022) 180:30–9. doi: 10.1016/j.theriogenology.2021.12.011 34952390

[30] Liu H Song X Huang M Zhan H Wang S Zhu S. Ureaplasma urealyticum induces polymorphonuclear elastase to change semen properties and reduce sperm motility: a prospective observational study. J Int Med Res (2022) 50:3000605221106410. doi: 10.1177/03000605221106410 35701892 PMC9208062

[31] Liu H Yang K He L Zhu S Pang T Zhu Z. Risk prediction of ureaplasma urealyticum affecting sperm quality based on mathematical model and cross-sectional study. Comput Math Methods Med (2022) 2022:2498306. doi: 10.1155/2022/2498306 35664640 PMC9159871

[32] Makarounis K Leventopoulos M Georgoulias G Nikolopoulos D Zeginiadou T Xountasi M. Detection of chlamydia trachomatis inside spermatozoa using flow cytometry: Effects of antibiotic treatment (before and after) on sperm count parameters. J Microbiol Methods (2022) 203:106604. doi: 10.1016/j.mimet.2022.106604 36330892

